# Comparative Analysis of Ectodermal Marker Expression in Human Adipose-Derived Stem Cells and Amniotic Epithelial Cells Exposed to Ectoderm-Inducing Conditions

**DOI:** 10.3390/ijms27114976

**Published:** 2026-05-30

**Authors:** Bartosz Sikora, Aleksandra Skubis-Sikora, Marcin Ciekalski, Patrycja Wieczorek, Agnieszka Prusek-Kucharek, Piotr Czekaj

**Affiliations:** 1 Department of Cytophysiology, Faculty of Medical Sciences in Katowice, Medical University of Silesia in Katowice, 40-055 Katowice, Poland; 2 Students Scientific Society, Department of Cytophysiology, Faculty of Medical Sciences in Katowice, Medical University of Silesia in Katowice, 40-055 Katowice, Poland

**Keywords:** stem cell plasticity, multipotent cells, adipose-derived stem cells, human amniotic epithelial cells, regenerative medicine, ectodermal differentiation

## Abstract

Nervous system and corneal disorders are major causes of permanent disability worldwide, largely due to the limited regenerative capacity of ectoderm-derived tissues. Therefore, the development of accessible and ethically acceptable cell-based therapies promoting the repair and regeneration of these tissues is of considerable translational importance. In this study, we aimed to comparatively evaluate the ectodermal differentiation potential of human adipose-derived stem cells (ADSCs) and human amniotic epithelial cells (hAECs) in vitro, with hAECs serving as a reference cell population with established ectodermal plasticity. Primary ADSCs and hAECs were characterized phenotypically using flow cytometry and functional differentiation assays. Cells were subjected to a directed ectodermal differentiation protocol and assessed via morphological analysis, immunostaining for ectoderm-associated proteins, and RT-qPCR analysis of lineage-specific genes. ADSCs exhibited morphological changes following differentiation, including a more epithelial-like phenotype and an increased nucleus-to-cytoplasm ratio. Immunostaining revealed the induction of nestin and *OTX2* expression after differentiation, which was particularly pronounced in ADSCs. Gene expression analysis demonstrated statistically significant upregulation of the ectoderm-related genes *EN2*, *SOX1*, and *PAX6* exclusively in hAECs. Results suggest that in ADSCs the differentiation process was only partially activated. In conclusion, our findings further support the suitability of hAECs as a reference cell line for studies investigating ectodermal differentiation protocols, while also demonstrating that ADSCs exhibit a limited but detectable capacity for acquiring ectoderm-specific characteristics under defined in vitro culture conditions.

## 1. Introduction

Nervous tissue and corneal impairments are among the leading causes of disability worldwide [1,2]. Due to the limited regeneration capacity of the central nervous system (CNS), damage leads to permanent structural and functional deficits. Moreover, advanced stages of severe corneal diseases require transplantation, but a shortage of donors prolongs the waiting time for surgery. Finding new sources of cell lines and investigating robust methods of cell differentiation are among the first steps toward the development of tissue structures that could be used for therapeutic purposes such as the regeneration of the CNS or the ocular surface. Successful differentiation of readily accessible stem cells toward the ectoderm lineage would pave the way for new cell therapies targeting, for example, the CNS or ocular surface.

To date, all approaches for differentiating cells from various sources possess both advantages and limitations, making them more or less suitable depending on the intended application. The selection of an appropriate cell source is a crucial factor in translating findings from basic research into clinical practice [3,4]. Adipose tissue is a source of mesenchymal stem cells (MSCs). Human adipose-derived stem cells (ADSCs) can be easily obtained through the enzymatic digestion of tissue samples, which can be taken from the patient. One method that provides minimal discomfort to the patient is liposuction. Several studies have confirmed ADSCs’ usefulness in tissue regeneration through direct cell application or via their paracrine activity, including the secretion of compounds or particles, such as extracellular vesicles [5,6,7]. Another promising candidate is the amniotic membrane. It is a joint source of distinct cell populations, including amniotic epithelial cells (hAECs) and amniotic membrane-derived mesenchymal stromal cells (AM-MSCs or AMSCs) [8,9,10,11,12,13]. Obtaining such tissues for further processing poses no health risk to the patient, mother or fetus, making them an accessible source of cells without ethical concerns. Both methods of tissue gathering create the possibility of generating personalized medicinal products and provide an easy source of allogenic stem cells when needed [4].

Regarding experimental cell identification protocols, ADSCs are required to adhere to plastic cell culture dishes, express MSC-specific antigens, and demonstrate the capacity to differentiate into mesodermal cell lines [14]. Commonly evaluated hAEC characteristics include the expression of epithelial tissue-specific cytokeratin proteins, pluripotent markers, and human leukocyte antigen proteins, as well as a cuboidal morphology and a lack of mesenchymal molecular markers [15]. Applying functional tests for hAEC identification is not common practice and none are recommended by the scientific community [16]. While hAECs are good candidates for cell-based tissue engineering, the specificity of their source makes these cells unavailable for autologous treatment, which in part underlines the need for investigating alternative solutions. It has been highlighted that both ADSCs and hAECs can acquire out-of-lineage phenotype characteristics under certain conditions of differentiation induction [17,18,19]. Moreover, hAECs are recognized as a cell population that contains a variable proportion of cells expressing pluripotent markers, thereby suggesting their pluripotency [20]. Studies have shown the broad plasticity and potential clinical applicability of both ADSCs and hAECs. Many reports have confirmed their ability to differentiate into cells of different lineages such as endoderm-originating hepatocytes, mesodermal tendon-like cells or osteoblast-like cells, and ectoderm-originating neural-like cells or corneal epithelial-like cells [20,21,22,23,24,25,26,27]. Nevertheless, only a limited number of studies have directly investigated the ectodermal differentiation of ADSCs using a representative cell line with confirmed ectodermal differentiation potential as a benchmark. Moreover, to the best of our knowledge, no previous study has directly compared the ectodermal differentiation potential of ADSCs and hAECs, suggesting that our study may be the first to address this issue.

This article presents an analysis comparing the ability of ADSCs and hAECs to differentiate into ectoderm-derived cell types, using hAECs as a control cell line due to their similar properties and well-established ectodermal differentiation protocol. The results indicate that ADSCs undergo morphological changes and partially activate the expression of ectodermal molecular markers in response to differentiating conditions in vitro.

## 2. Results

### 2.1. Cell Characteristics

**Figure 1:**
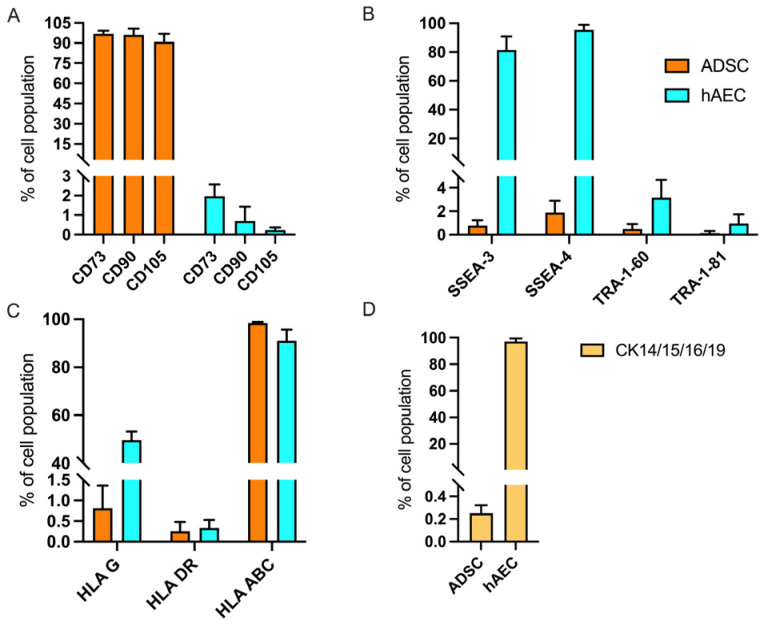
Cell identification via flow cytometry. (**A**) The percentage of cells positive for CD73, CD90, and CD105 antigens. (**B**) The percentage of cells positive for pluripotent markers SSEA-3, SSEA-4, TRA-1-60, and TRA-1-810. (**C**) The percentage of cells positive for HLA-G, HLA-DR, and HLA-ABC. (**D**) The percentage of cells positive for CK14/15/16/19. Bars represent the means ± standard deviations (SDs).

The mesenchymal phenotype of the ADSCs and the epithelial phenotype of the hAECs were confirmed (Figure 1). Both cell lines expressed MHC I proteins at low levels and did not express MHC II proteins. ADSCs expressing the pluripotent markers at a low level were considered negative. In contrast, the SSEA-3 and SSEA-4 antigens were expressed in hAECs; however, TRA-1-60 and TRA-1-81, which are specific to embryonic cells, were expressed at very low levels. These findings are consistent with our previous report [28].

Surface markers specific for mesenchymal stem cells, CD73, CD90, and CD105, were analyzed in both cell lines prior to differentiation (Figure 1A). The majority of the ADSC population expressed CD73 (mean 96.8% ± 2.38), CD90 (mean 96.06% ± 4.69), and CD105 (mean 90.91% ± 5.88) in all examined samples. In contrast, hAECs showed barely detectable expression of CD73 (mean 1.97% ± 0.59), CD90 (mean 0.71% ± 0.7), and CD105 (mean 0.24% ± 0.13), levels that are considered negative for these markers. Next, cells were assessed based on the expression of human leukocyte antigen (HLA) system proteins, specifically HLA type G and HLA type ABC (MHC class I), along with HLA type DR (MHC class II) (Figure 1C). We detected low levels of HLA G (mean 0.81% ± 0.55) and HLA DR (mean 0.25% ± 0.23) proteins in ADSCs, both considered negative. Most of the ADSC population expressed HLA ABC proteins (mean 98.41% ± 0.5). Half of the hAEC population (mean 49.69% ± 3.5) expressed HLA-G and most of the population (mean 91.07% ± 4.58) expressed HLA ABC. The HLA-DR proteins were detected at low levels (mean 0.33% ± 0.2), which is considered negative. Proteins specific to pluripotent cells were analyzed in both cell lines. ADSCs did not express SSEA-3 (mean 0.79% ± 0.47), SSEA-4 (mean 1.88% ± 1.03), TRA-1-60 (mean 0.5% ± 0.4), or TRA-1-81 (mean 0.14% ± 0.18). The hAECs presented high levels of SSEA-3 (mean 75.18% ± 3.05) and SSEA-4 (mean 95.38% ± 3.54). Similar to the ADSCs, we observed negligible expression of TRA-1-60 (mean 3.17% ± 1.5) and TRA-1-81 (mean 0.97% ± 0.77) in hAECs (Figure 1B). The epithelial cell phenotype was investigated in both cell lines via the detection of cytokeratin 14, 15, 16, and 19 (Figure 1D). The level of cytokeratins expressed in ADSCs was low (mean 0.25% ± 0.07), considered negative, and high (mean 97.22% ± 2.12) in hAECs.

### 2.2. Multipotency Assessment

**Figure 2:**
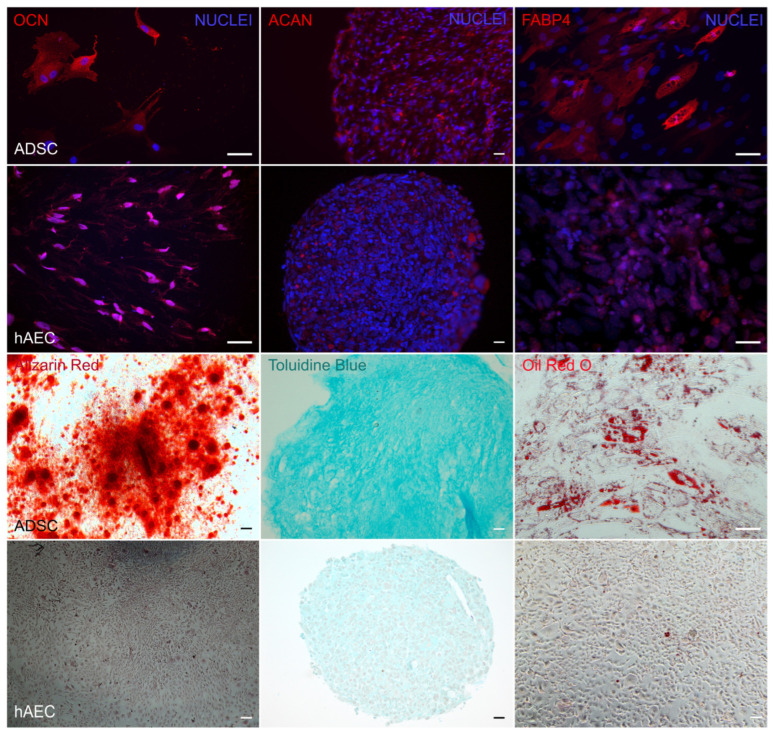
Multipotency assessment of ADSCs and hAECs. Upper panel—immunofluorescent detection of osteocytes with anti-OCN (osteocalcin), anti-ACAN (aggrecan), and anti-FABP4 (fatty acid binding protein 4) antibodies. Cell nuclei were counterstained with DAPI. Lower panel—cytochemical staining of calcium deposits (Alizarin Red S), acid mucopolysaccharides (Toluidine Blue), and lipid droplets (Oil Red O); scale bars represent 15 µm.

ADSCs successfully differentiated and produced lipid droplets, calcium deposits, and acid mucopolysaccharides. Differentiation of hAECs was unsuccessful for adipocytes and poor for osteocytes (Figure 2).

### 2.3. Ectodermal Differentiation

Both cell lines underwent differentiation into ectoderm-like cells over a 7-day period, which was assessed via morphological examination and by examining the expression of ectodermal-specific proteins and genes.

#### 2.3.1. Morphology Assessment

**Figure 3:**
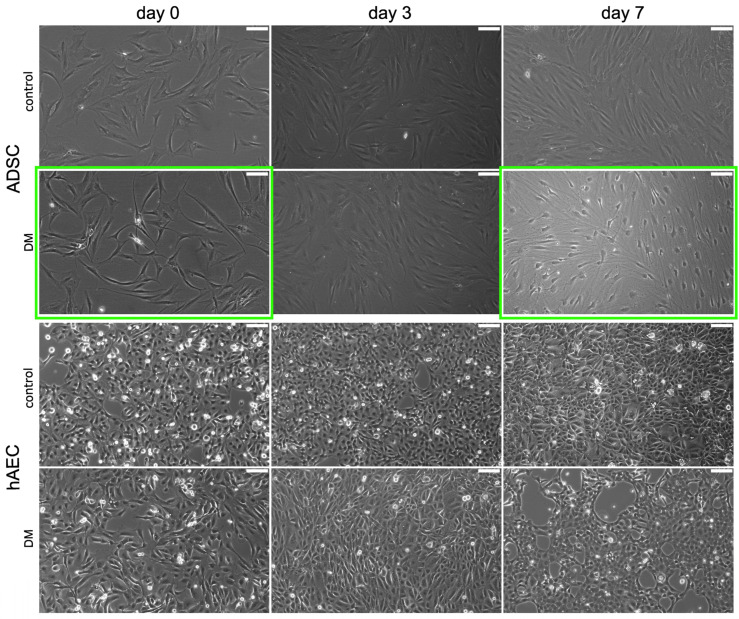
Morphology of ADSCs and hAECs subjected to ectoderm-differentiating medium (DM). Scale bar: 15 µm. Green frames indicate morphology change between differentiated and undifferentiated ADSCs.

The cell morphology of ADSCs significantly altered after differentiation (Figure 3). The cells developed a rounder shape, had denser nuclei and cytoplasm, and showed an increase in nucleus-to-cytoplasm (N/C) ratio. In contrast, no prominent change was seen in the morphology of hAECs. The cells remained round and maintained their N:C ratio.

#### 2.3.2. Immunostaining of Ectoderm-Specific Proteins

**Figure 4:**
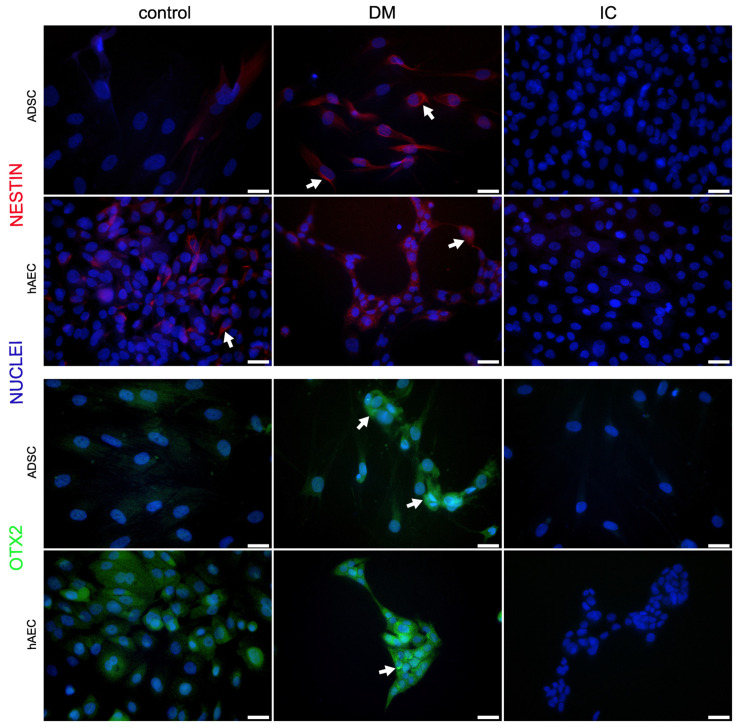
Detection of nestin and *OTX2* in ADSCs and hAECs in cell culture with ectoderm differentiation medium (DM) in comparison with control condition (standard culture medium without differentiation factors). Red, nestin; blue, nuclei; green, *OTX2*; IC, isotype control; 40× magnification. Scale bar: 15 µm. Arrows indicate the examples of positive signals.

After differentiation, nestin was expressed in ADSCs (ADSC—DM), but it was not detected in control cells (ADSC—control). Nestin expression in hAECs was observed in both the study and control group. *OTX2* was identified after differentiation in both cell lines (Figure 4).

#### 2.3.3. Ectoderm-Specific Gene Expression Analysis

**Figure 5:**
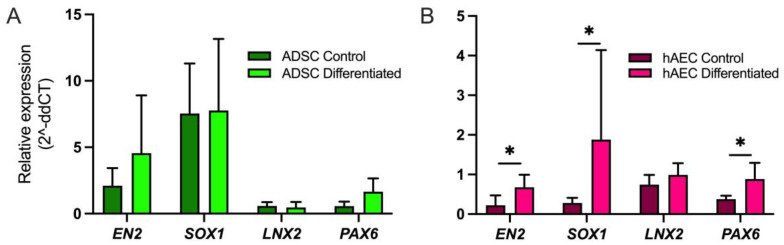
*EN2*, *SOX1*, *LNX2*, and *PAX6* expression after ectodermal differentiation. (**A**) ADSCs. (**B**) hAECs. * *p* < 0.001 vs. control (Student’s *t*-test).

After differentiation, ADSCs and hAECs were evaluated based on the expression of ectoderm-specific genes—specifically, *EN2*, *SOX1*, *LNX2*, *PAX6*, and *PPIH*—as an endogenous control. ADSCs showed higher expression of *EN2* (1.7-fold higher), *SOX1* (1.2-fold higher), and *PAX6* (2,4-fold higher) in comparison with control cells. The expression of *LNX2* appeared similar to the control cells, but slightly lower. However, the observed changes were not large enough to be statistically significant (Figure 5A). In hAECs, we noticed a higher expression of *EN2* (4.5-fold higher), *SOX1* (4.4-fold higher), and *PAX6* (2.3-fold higher), and these changes were statistically significant (Student’s *t*-test, *p* < 0.001). Similar to the ADSCs, *LNX2* expression was comparable with the control; although slightly higher after differentiation, the change was not large enough to be statistically significant (Figure 5B). This suggests that ectodermal pathways are activated in response to the applied protocol.

## 3. Discussion

Fundamental studies employing primary stem cell lines require thorough characterization of the cells to confirm their true phenotype. In this study, we initially assessed both ADSCs and hAECs based on the expression of mesenchymal, epithelial, and pluripotent markers. The results confirmed the mesenchymal phenotype of ADSCs, as well as the epithelial phenotype of hAECs. Moreover, low levels of TRA-1-60 and TRA-1-81 expression confirmed the persistence of embryonic molecular markers in isolated hAECs and suggest that these cells might partially retain their pluripotent phenotype [28].

Since the multipotency of ADSCs must be assessed to confirm their identification, we subjected both cell lines to osteo-, chondro-, and adipogenic differentiation to complete the step-by-step methodology and expose potential differences between them. The ADSCs successfully differentiated into targeted cell lineages, finally confirming the initial cell identification. In contrast, we did not recognize any positive staining for lipid droplets or fatty acid binding proteins in hAECs after differentiation. This finding may raise questions about the plasticity of hAECs, which are recognized as pluripotent-like cells [15,21,29]. However, our observations correspond to those reported in the study of Díaz-Prado et al. (2010), who showed that mesodermal cell lineage differentiation is more effective for AM-MSCs than hAECs and reported that hAECs possess the capacity to differentiate into osteocytes and chondrocytes, but not adipocytes [9]. Similarly, Si et al. (2015), in their study on the osteogenic potential of stem cells from different sources, compared hAECs with MSCs from bone marrow (BMMSCs) and alveolar cleft [30]. They found that the osteogenic potential of hAECs was significantly lower than that of MSCs [30]. To confirm our observation, we performed functional differentiation of hAECs in three separate experiments. The cells consistently failed to differentiate into adipocytes; however, osteo- and chondrogenesis occurred, albeit less efficiently than in ADSCs.

After the initial cell phenotype assessment, we proceeded with ectodermic differentiation, which appeared successful. To induce ectodermic differentiation, we used a commercially available set of chemicals. We visualized the results with light and fluorescent microscopy. The first revealed that ADSCs, in differentiating media, developed a rounder and more condensed shape, appearing similar to cobblestones, as is characteristic of epithelial cells. The immunostaining of ectodermal-specific molecular markers such as nestin and *OTX2* showed positive signals under a fluorescence microscope. Together, these visual observations showed that the ADSCs acquired ectoderm-specific attributes. In comparison, the hAECs did not show prominent changes in their morphology. Although the cells expressed nestin and *OTX2*, the former was also present in undifferentiated cells, which is characteristic of cells of this origin (at a low level) [21,31]. We evaluated the microscopic observations—obtained by analyzing the selected molecular markers via RT-qPCR—and found changes in levels of *EN2*, *SOX1*, and *PAX6* mRNAs. The expression levels of the examined genes were normalized to the *PPIH* housekeeping gene, which had been selected based on a preliminary assessment previously conducted in our department. The stability of *PPIH* (Peptidylprolyl Isomerase H) expression was compared with that of *ACTB* (Beta-Actin) and *GAPDH* (Glyceraldehyde-3-Phosphate Dehydrogenase), and only *PPIH* demonstrated stable expression in both cell lines.

The function of the *EN2* gene is commonly linked to mid-brain development [32]. Our findings show that the expression of *EN2* was upregulated in both cell lines after differentiation; however, the change was statistically significant only in the hAEC group. Similarly, Trzaska et al. (2007) showed its increased expression in bone-marrow-derived MSCs after neurogenic differentiation [33]. As reported by Miwa and Era (2018), Sox1 is known to be the most specific marker for neuroepithelial cells [34]. Their study confirmed the ectodermal origin of MSCs; however, Sox1-traced MSCs showed that most of the Sox-1 positive cells present in adipose tissue originate from the bone marrow [34]. Morys et al. (2022) examined the influence of *NGF-β* overexpression in MSCs derived from Wharton’s jelly on neural pathway gene activity, with a particular focus on *SOX1* expression [35]. They suggested that overexpressing *NGF-β* may drive MSCs to differentiate into neural lineages [35]. In relation to our observations, *SOX1* gene expression remained unchanged after differentiation, and although an upregulation occurred, it was not large enough to be statistically significant. Moreover, their study confirmed upregulated expression of *NES*. In our study, its expression was confirmed by fluorescence microscopy.

The study of Liu et al. (2023) showed that *LNX2* may contribute to the neuronal differentiation of ADSCs via activation of the wnt/β-catenin signaling pathway [36]. The *LNX2* gene product is widely expressed in several adult tissues, including the CNS from the earliest stages of embryonic development. In their study, it was reported that *LNX2* expression was upregulated after the neural differentiation induction of ADSCs [36]. Our protocol did not induce distinctive changes in *LNX2* expression in either tested cell line. A slight upregulation was observed in hAECs, but it was not statistically significant.

Dos Santos et al. (2019) used *PAX6* expression analysis to assess the progression of keratinocyte differentiation in ADSCs and BMMSCs [37]. The study results showed that *PAX6* expression increased in both cell lines after 7 days of differentiation [37]. Similar to our study, Brown et al. (2022) differentiated umbilical cord-derived MSCs into retinal pigment epithelium-like cells, showing that the expression of *NES*, *PAX6*, and *OTX2* was upregulated after differentiation [38]. We confirmed the presence of *NES* and *OTX2* by fluorescence microscopy, and observed an upregulation of *PAX6*, but this was not statistically significant.

This study has several limitations. Despite the positive fluorescence signals, the RT-qPCR results did not confirm these observations due to the low expression of ectoderm-specific molecular markers in ADSCs, suggesting that the protocol was not efficient for these cells. Nevertheless, the slight effects we observed may be attributable to the relatively short ectodermic differentiation induction. For example, it was previously reported that the expression of ectodermic markers such as *EN2* depends on differentiation time in MSCs and appears stronger after 12 days of differentiation in comparison with the 6-day-long incubation results [33]. Based on our findings, it might be assumed that the ectodermic pathways have been activated but the study does not provide definitive evidence for successful differentiation. However, it should be noted that the time of differentiation which we have applied in our study was based on the manufacturer’s suggestion disclosed in the technical protocol. The time of differentiation usually is one of the key factors influencing the activation gene promotors. It should be optimized for each experimental approach of this kind, particularly in the context of patient-related variability of primary cell lines. Additionally, the results of gene expression assessment should be verified via proteomic analysis. It is known that changes in gene expression do not always correspond to protein levels, and conclusions drawn solely from RT-qPCR may be incomplete. Gene silencing, potentially triggered by feedback mechanisms, could also contribute to this discrepancy [39]. Moreover, although our selection of molecular markers for ectodermal differentiation included key indicators commonly used in this field, the panel may still be considered limited. A more in-depth investigation of ectodermal differentiation pathways could therefore provide a more comprehensive explanation of the observed effects.

Our findings may contribute to the advancement of the current field by providing new insights into ADSC plasticity, as well as a comprehensive analysis of hAECs. This study also presents a step-by-step characterization approach for primary stem cells, which should be carefully performed prior to experimental procedures in order to confirm cell phenotype and ensure the reliability of the obtained results and subsequent conclusions. The methodology summarized here is consistent with current recommendations of the scientific community and relevant scientific societies and may serve as a useful framework for future studies. Regarding future perspectives and potential clinical translation, it may be valuable to investigate pre-transplantation stem cell priming using tailored culture media to activate specific molecular pathways. Such an approach could potentially improve stem cell adaptation to the host environment and enhance tissue integration and regenerative outcomes.

## 4. Materials and Methods

### 4.1. Experimental Design

In this in vitro study, we investigated normal human ADSCs and hAECs. Collection of tissue samples was approved by the local bioethics committee (decision numbers PCN/CBN/0052/KB/208/22 and PCN/CBN/0052/KB/211/22). Prior to the experiment, both primary cell lines were separately tested for Mycoplasma spp. and assessed based on their phenotype and quality. Viable and healthy cells were selected to undergo the ectodermal differentiation protocol and the evaluations of its outcome. The effectiveness of differentiation was assessed using morphological analysis, immunostaining, and gene expression analysis. The study groups were compared with applied controls and assessed statistically.

### 4.2. Tissue Collection

Fat from subcutaneous abdominal adipose tissue was acquired through liposuction from three healthy female patients during procedures conducted in Mazan Surgical Clinics in Katowice. The tissue was collected and used for these experiments after obtaining written informed consent from the patients. The human placentas were obtained from three normal, full-term pregnancies delivered by cesarean section for obstetric reasons, i.e., transverse position of the fetus, longitudinal pelvic position, and fetopelvic disproportion. The tissue was collected and used for the experiments after obtaining written informed consent from patients admitted to the Department of Gynaecology and Obstetrics in the Division of Gynaecologic Oncology at the Brothers Hospitallers Hospital in Katowice.

### 4.3. Adipose-Derived Stem Cell Isolation

Adipose tissue samples were minced and digested with collagenase type I (Lonza, Basel, Switzerland) for 2 h at 37 °C with orbital shaking. The enzyme activity was stopped by adding cell culture medium containing fetal bovine serum (FBS, Euroclone, Pero, Italy). The digested tissue portions were centrifuged at 500× *g* for 5 min to pellet down the stromal vascular fraction (SVF) and separate the cells from the remaining tissue fragments and cell medium. The supernatant was subsequently discarded. Then, the SVF pellet was resuspended in a cell culture medium, transferred into culture dishes, and incubated for 48 h. Adherent cells were rinsed with PBS to discard residing dead cells and tissue debris and passaged when they reached 70% confluence.

### 4.4. Human Amniotic Epithelial Cell Isolation

The amniotic membrane was manually separated from the chorion and then cut into pieces. The pieces were enzymatically digested with trypsin (0.05%) supplemented with EDTA (Gibco, Grand Island, NY, USA) for 40 min at 37 °C with rotation. After digestion, the cell suspension was centrifuged at 500× *g* for 5 min at 4 °C. The pellet was resuspended in culture medium, then transferred into culture dishes and incubated overnight.

### 4.5. Cell Culture Conditions

ADSCs were maintained as control cells in DMEM (Dulbecco’s Modified Eagle Medium, Gibco, Grand Island, NY, USA) supplemented with 10% of fetal bovine serum (FBS, EuroClone, Pero, Italy), 1% antibiotic–antimycotic mixture (Gibco, Grand Island, NY, USA), and 1% Glutamax solution (Gibco, Grand Island, NY, USA) at 37 °C in a 5% CO_2_ incubator (Sanyo MCO-19M, Osaka, Japan). As for the hAECs, they were maintained as control cells in DMEM with L-glutamine (Dulbecco’s Modified Eagle Medium, Coring, Corning, NY, USA), supplemented with 10% of FBS (EuroClone, Pero, Italy), 1% antibiotic–antimycotic mixture (Gibco, Grand Island, NY, USA), and 10 ng/mL of EGF (Thermo Fisher, Waltham, MA, USA) at 37 °C in a 5% CO_2_ incubator (Sanyo MCO-19M, Osaka, Japan). The culture medium was changed every 2–3 days. Cells were assessed with an Olympus IX73 microscope (Olympus, Shinjuku, Tokyo, Japan) and a CMOS camera (Hamamatsu, Hamamatsu, Japan) was used for photographic documentation. ADSCs were used after the 2nd passage and hAECs were used after the 1st passage. The cells were cultured in 24-, 12-, and 6-well plates and T25 flasks, according to the specific requirements of each test.

### 4.6. Initial Phenotype Evaluation with Flow Cytometry

ADSCs and hAECs were analyzed cytometrically to assess the expression of characteristic markers for mesenchymal and epithelial cells, pluripotent markers, and major histocompatibility complex (MHC) class I and II. Specifically, the markers used were as follows: Mesenchymal stem cell molecular markers—positive: CD90, CD73, and CD105; negative: CD45, CD34, CD11b, CD79A, and HLA-DR. Pluripotent markers: SSEA-4, SSEA-3, TRA-1-60, TRA-1-81. Epithelial cell markers: cytokeratin 14, 15, 16, and 19. Human leukocyte antigen (HLA) system molecular marker panel: HLA-ABC, HLA-G, major MHC class I, HLA-DR, DP, DQ, and major MHC class II. The antibody set used for the analysis is presented in Table 1. Cell fluorescence was measured immediately after staining with Cytoflex Flow Cytometer (Beckman Coulter, Brea, CA, USA) and data were analyzed using software CytExpert (Beckman Coulter, Brea, CA, USA). Results are presented as counts per 10,000 events.

### 4.7. Multipotency

ADSCs and hAECs underwent functional identification to assess their multipotency. The test was performed with cytochemical and immunostaining methods following the functional assessment of their ability to differentiate into adipocytes, chondrocytes, and osteocytes using a Human Mesenchymal Stem Cell Functional Identification Kit (Cat. #SC006, R&D Systems, Minneapolis, MN, USA). For adipogenesis, the cells were cultured in basal medium with adipogenic supplement (containing hydrocortisone, isobutylmethylxanthine, and indomethacin); for osteogenesis, the cells were cultured in basal medium with osteogenic supplement (containing dexamethasone, ascorbate phosphate, and β-glycerol phosphate); and for chondrogenesis, cells were cultured with an addition of chondrogenic supplement (containing dexamethasone, ascorbate phosphate, proline, pyruvate, and recombinant TGF-β3). Adipogenesis and osteogenesis were induced in adherent cells growing in a monolayer. For chondrogenic differentiation, the cells were cultured in a conical tube as a pellet. The differentiating medium (DM) was changed every 3–4 days. The cell culture was conducted for 7–21 days according to the manufacturer’s protocol. The lipid droplets were stained with oil red O solution (01391-250ML, Sigma–Aldrich, St. Louis, MO, USA), the calcium deposits were stained with Alizarin red solution (2003999, Sigma–Aldrich, St. Louis, MO, USA), and the acid mucopolysaccharides were stained with Alcian Blue staining solution (TMS-010-C, Sigma-Aldrich, St. Louis, MO, USA) accordingly. Additionally, FABP4 (fatty acid binding protein 4), osteocalcin, and aggrecan were detected with antibodies (Table 1). The anti-FABP4 and anti-aggrecan antibodies were included in the Human Mesenchymal Stem Cell Functional Identification Kit and were used according to the manufacturer’s protocol. The antibodies were detected using a secondary donkey anti-goat IgG or goat anti-mouse IgG antibody (Table 1). The cell nuclei were counterstained with DAPI (H-1500, Vector, Newark, CA, USA).

### 4.8. Differentiation into Ectoderm-like Cells

ADSCs and hAECs were cultured in differentiating media supplied with the STEMdiff Trilineage Differentiation Kit (05230, Stem Cell Technologies, Vancouver, BC, Canada), which was designed for differentiating human embryonic stem cells (ESCs) and iPSCs into all three germ layers. Here, only the ectoderm medium kit component was used, which is a complete medium to perform in vitro directed differentiation into ectoderm-like cells. Cells were plated, and after 24 h, the standard medium (SM/control) was replaced with ectoderm-differentiating medium (DM) and the cells were cultured for 7 days with daily medium changes. Subsequently, cells were either harvested or fixed for the analysis of lineage-specific markers using immunostaining or transcriptome analysis via RT-qPCR. Moreover, changes in cell morphology were documented every day for 7 days of differentiation.

### 4.9. Immunodetection of Ectoderm-Specific Proteins

After differentiation, cells were fixed with 4% formaldehyde for 15 min. This was followed by permeabilization with 0.5% Triton X-100 solution. Nonspecific protein binding was blocked by incubating the samples in PBS with 1% bovine serum albumin (BSA) for one hour at room temperature. Nestin and *OTX2* were detected in ADSCs and hAECs with monoclonal antibodies (Table 1). The control cells were not subjected to differentiation and were cultured simultaneously in the standard medium. Samples were incubated with a primary anti-nestin antibody (1:50) detected with an Alexa Fluor 568 fluorochrome-conjugated secondary antibody (1:1000), and with a primary anti-OTX2 antibody (1:2000) detected with an Alexa Fluor 488 fluorochrome-conjugated secondary antibody (1:1000). Cell nuclei were visualized with DAPI (Vectashield Vibrance Antifade Mounting Medium, H-1700, Vector, Newark, CA, USA). The isotype control for nestin was anti-mouse IgG1 and for *OTX2*, anti-rabbit IgG.

### 4.10. Expression of Genes Related to the Ectoderm Cell Lineage

Total RNA was extracted from cells using TRIzol reagent (Invitrogen, Carlsbad, CA, USA) according to the manufacturer’s instructions. The RNA concentration was determined using a Nanodrop 2000 spectrophotometer (Thermo Fisher, Waltham, MA, USA) and the quality of RNA was analyzed with agarose electrophoresis gel. Gene expression assessments of *EN2*, *SOX1*, *LNX2*, *PAX6*, and *PPIH* were carried out using real-time RT-qPCR with SYBR Green chemistry (GoTaq 1-Step RT-qPCR System, A6020, Promega, Madison, WI, USA) and Light Cycler 96 Instrument (Roche, Basel, Switzerland). All samples were tested in three biological and two technical replicates (n = 6). *PPIH* served as an endogenous positive control (a housekeeping gene) of amplification. Pre-designed oligonucleotide primers were acquired commercially (Sigma–Aldrich, St. Louis, MO, USA). Details regarding the primer set can be found in Table 2. Reactions were completed using melting curve analysis to confirm the specificity of amplification and the absence of primer dimers.

### 4.11. Statistical Analysis

All statistical computations were performed in Statistica 13.3 software (TIBCO Software Inc., Palo Alto, CA, USA). Study groups were compared using Mann–Whitney U-tests for non-normally distributed data and Student’s *t*-tests for normally distributed data. The level of significance was set at 5% (α = 0.05). Values for non-normally distributed data are presented as medians with 25th and 75th quartiles, as well as the minimum and maximum values. Normally distributed data are presented as means and standard deviations (SDs).

## 5. Conclusions

In summary, our study provides new insights into the ectodermal differentiation potential of ADSCs through direct comparison with hAECs. We demonstrated that both cell lines expressed ectodermal markers following induced ectodermal differentiation, although the overall efficiency of the process was moderate. We also observed that ADSCs underwent morphological changes toward a more epithelial-like phenotype in response to the applied differentiation conditions. Furthermore, only hAECs exhibited increased expression of ectoderm-specific genes, suggesting that in ADSCs, the differentiation process was only partially activated. These findings indicate that the applied protocol requires further refinement to improve the efficiency of ectodermal differentiation in ADSCs. Our results also support the suitability of hAECs as a reference cell line for studies investigating ectodermal differentiation protocols. Moreover, adipose tissue may represent a feasible and accessible source of cells capable of acquiring ectoderm-specific characteristics under defined in vitro conditions.

## Figures and Tables

**Table 1:** The set of antibodies used for target protein detection.

**Conjugated**								
Application	Target protein-fluorophore, Cat. #			Isotype control, Cat. #			dilution factor	Producer
MSC markers	CD90-APC IgG_2A_ ^1^			IgG_2A_-APC ^1^			1:100	R&D, USA
	CD73-CFS IgG_2B_ ^1^			IgG_2B_-CFS ^1^				
	CD105-PerCP IgG_1_ ^1^			IgG_1_-PerCP ^1^				
	CD45-PE IgG_1_ ^1^			IgG_1_-PE ^1^				
	CD34-PE IgG_1_ ^1^			IgG_2B_-PE ^1^				
	CD11b-PE IgG_2B_ ^1^			-				
	CD79A-PE IgG_1_ ^1^							
	HLA-DR-PE IgG_1_ ^1^							
Pluripotency markers	SSEA-4-FITC, 560126			IgG_3_-FITC, 555578			1:20	BD, USA
	SSEA-3-PE, 560237			IgG_1_-PE, 555749				
	TRA-1-60-Cy5.5, 561573			IgM-Cy5.5, 560857				
	TRA-1-81-AF700, FAB8495N			IgM- AF700, IC015N				R&D, USA
Epithelial cell markers	Cytokeratin 14–16, 19 set-PE, 550953			IgG_1_-PE, 550953			1:5	BD, USA
HLA system	HLA-ABC-APC, 555555			IgG_1κ_-APC, 555751				
	HLA-DR, DP, DQ-FITC, 555558			IgG_2a,κ_-FITC, 555573				
	HLA G-PE, ab24384			IgG_1_-PE, ab91357			1:100	Abcam, UK
**Unconjugated**								
Application	Target protein, Cat. #	(I, II, IC) ^3^	fluorophore		dilution factor	host	target	Producer
MSC functional verification	FABP4, 967799 ^2^	I	-		1:50	goat	human	R&D, USA
	Aggrecan, 967800 ^2^	I	-		1:200	mouse	human	
	IgG, NL001 ^2^	II	NL557		1:1000	donkey	goat	
	Osteocalcin, ab13420	I	-		1:200	mouse	human	Abcam, UK
	IgG, ab175473	II	AF568		1:1000	goat	mouse	
Ectodermic differentiation evaluation	Nestin, sc-23927	I	-		1:50	mouse	human	SCBT, USA
	IgG, ab175473	II	AF568		1:1000	goat	mouse	Abcam, UK
	OTX2, 13497-1-AP	I	-		1:200	rabbit	human	Proteintech, USA
	IgG, ab150077	II	AF488		1:1000	goat	rabbit	Abcam, UK
	IgG1, ab170190	IC	-		1:50	mouse	yeast	
	IgG, 3900	IC	-		1:200	-	rabbit	Cell Sign., USA

^1^ Components of “Mesenchymal Stem Cell Verification Flow Kit” (Cat. #FMC020); ^2^ Components of “Human Mesenchymal Stem Cell Functional Identification Kit” (Cat. #SC006); ^3^ I-primary antibody/II-secondary antibody/IC-isotype control.

**Table 2:** Primer sequences used for RT-qPCR.

*Target*	*Description*	*Primer*	*Sequence (5′–3′)*	*Product Size [bp]*	*Tm [°C]*	*GenBank Accession Numbers*
*PPIH*	Reference gene	Forward	ATCATCGATGGACTTCTAGTG	119	82.5	NM_001330510.2
		Reverse	AGTCTTTGTCTGGACTACATC			
*EN2*	Ectoderm-specific genes	Forward	GTCTGAATAACCATCTGCTG	81	78.8	NM_001427.4
		Reverse	AAAACAGAAACCAACTTTGC			
*SOX1*		Forward	TGCTTGTTCTGTTAACTCAC	100	80.5	NM_005986.3
		Reverse	AAAGAACCTCAGAGAGAGTC			
*LNX2*		Forward	GAAAGATTTCTGTCCGTTGG	171	79.5	XM_054374212.1
		Reverse	CATCTGTTTTTGAGATGTGC			
*PAX6*		Forward	AGAGAATACCAACTCCATCAG	152	82.5	NM_001368912.2
		Reverse	GATAATGGGTTCTCTCAAACTC			

## Data Availability

All data are contained within the article. The original contributions presented in this study are included in the article. Further inquiries can be directed to the corresponding author.
